# The neurobiology of suicide - A Review of post-mortem studies

**DOI:** 10.1186/2049-9256-1-2

**Published:** 2013-04-23

**Authors:** Karolina Furczyk, Barbora Schutová, Tanja M Michel, Johannes Thome, Andreas Büttner

**Affiliations:** 1 Department of Psychiatry, University of Rostock, Gehlsheimerstrasse 20, 18147 Rostock, Germany; 2 College of Medicine, Swansea University, Singleton Park, Swansea, SA2 PP UK; 3 Institute of Forensic Medicine, University of Rostock, St.-Georg-Strasse 108, 18055 Rostock, Germany

**Keywords:** Autopsy, Biological Psychiatry, Depression, Genetics, Hypothalamic-pituitary-adrenal (HPA) axis, Neural Plasticity, Neurotransmitter, Proteomics

## Abstract

The neurobiology of suicidal behaviour, which constitutes one of the most serious problems both in psychiatry and general medical practice, still remains to a large degree unclear. As a result, scientists constantly look for new opportunities of explaining the causes underlying suicidality. In order to elucidate the biological changes occurring in the brains of the suicide victims, studies based on post-mortem brain tissue samples are increasingly being used. These studies employ different research methods to provide an insight into abnormalities in brain functioning on various levels, including gene and protein expression, neuroplasticity and neurotransmission, as well as many other areas. The aim of this paper to summarize the available data on the post-mortem studies, to provide an overview of main research directions and the most up-to-date findings, and to indicate the possibilities of further research in this field.

## Introduction

Suicide is one of the leading causes of death globally for all ages. Every year, nearly one million people die from suicide according to WHO, 2013. It therefore constitutes a serious medical and social problem, and as such calls for profound and systematic research to enable a better understanding of the underlying pathology, as well as the development of new therapeutic approaches. Suicides and suicide attempts are usually associated with mental illness including affective or psychotic disorders as well as alcohol and/or substance abuse disorders [1, 2]. Suicide is therefore an important focus of neuropsychiatric research. Among psychiatric disorders, mood disorders, schizophrenia and alcoholism are most often co-morbid with suicide [3]. A number of risk factors for suicidal behaviour has been identified, including stress [4], impulsive-aggressive behaviour [5], chronic disease [6] and hopelessness [7]. On the other hand, protective factors such as a stable relationship, a well-established social network and a dependable financial situation [8, 9] have also been described. While many socioeconomic correlates for suicide have been identified, the neurobiology of suicide remains less clear. The studies of biological abnormalities associated with suicidal behaviour, performed in biomaterials such as blood cells, cerebrospinal fluid and plasma obtained from suicidal patients, have shed some light on this matter. As a result, an involvement of serotonergic [10–13], dopaminergic and noradrenergic systems [14, 15], as well as abnormalities in the hypothalamic-pituitary-adrenocortical axis [14, 16] were proposed. However, it is not clear if, and to what extent, those observations reflect the pathomechanisms in the brain. Here, the availability of well-characterized post-mortem brain samples of suicide victims has proven very useful for research. This article gives a description of the nature of post-mortem studies, of the opportunities they provide, but also of their limitations. Next, it summarizes the results emerging from these studies and its role in enhancing our understanding of suicide. We have aimed at providing the reader with an overview of primary studies looking at genetics, proteomics, neurotransmitter systems, cell-signaling, neural plasticity and neuroendocrinology, that used specific and reproducible methods [17]. Overall, we aimed at summarizing consistent results, already described and evaluated by a number of different authors. On the other hand, studies showing contradictory results reflect the complexity of the presented problems. In order to better point out new research directions, such inconsistent studies have also been included in our review. Last but not least, although we concentrate on the most up-to-date findings, we also describe a number of the most important older studies, which have provided the basis for the more recent ones.

### Review

#### Post-mortem studies in suicide victims

Post-mortem brain samples obtained from suicide victims and controls offer many new opportunities to study molecular mechanisms underlying suicidal behaviour. In contrast to earlier studies based on peripheral tissues, research enables a direct insight into the neurobiological abnormalities associated with suicide. However, it is important to be aware that those studies need to meet certain criteria [18].

To begin with, a positive opinion of the local ethical commission is mandatory for every study involving the examination of human brain samples. Next, because the brain tissue degrades very easily, the process of collecting samples for post-mortem studies should be carried out with special care. The collection of the samples should ideally take place no longer than 48 hours after death. In order to establish the quality of collected tissue and determine whether it is adequate for measuring protein levels and gene expression, pH of the samples and the RNA integrity number should be determined. Since methyltransferase- and acetyltransferase-activities measured in brain samples were reported to be relatively preserved and independent of storage duration or post-mortem interval Monoranu et al. [19] proposed to use their levels as stability markers in epigenetic studies. On the other hand, an increased storage period has been shown to influence tryptophan levels, which led to the conclusion that the level of this amino acid might indicate the level of protein degradation in the sample [20]. Next, it is very important to carefully examine the samples for any neuropathological abnormalities. This can be combined with dissecting particular regions of the brain, so that the exact neuroanatomical location of examined molecular processes can be taken into account. Since many psychiatric patients have been on several medications, and a drug addiction is often associated with suicidality, it is also advisable to obtain an accurate toxicological screening of the samples. Alternatively, toxicological tests can be performed using the blood of the subjects. Last but not least, a “psychological autopsy” should be obtained from the subjects’ families and friends, including psychiatric diagnoses, physical disorders, childhood history, possibilities of early life trauma and history of adverse life events. Nowadays, post-mortem brain collection programs [21] have minimized the difficulties resulting from these prerequisites, readily offering samples collected using standardized methods. Studies based on brain tissue have been focusing different immunological and biochemical mechanisms, as well as gene expression and brain cytoarchitecture. Below are presented the key topics of this research in relation to suicidality.

### Genetic studies

Looking at a possible role of various genetic factors in suicidal behaviour can broaden our knowledge of different neurobiological elements responsible for this pathology. A number of studies have focused on one or several genes at a time, comparing suicide victims with controls. This led to the identification of genetic predisposing factors, such as WFS1 [22, 23] or p75NTR [24] as well as many others. On the other hand, many researchers proposed an involvement of a whole family of genes or certain pathways rather than just single genes in the pathology of different psychiatric disorders [25, 26]. For example, Must et al. [27] found common variations in a group of genes located in the 4p locus to be related to completed suicide in male individuals. However, to confirm the biological relevance of these studies, it is necessary to replicate the findings in similar and comparable clinical samples with comprehensive coverage of the variants at a particular candidate locus [28]. To this end, genome-wide association studies and mRNA expression studies can help to verify to the significance of previous reports and distinguish biochemical pathways and processes particularly important to the pathophysiology of suicide. The association studies provide a powerful tool for discovering the strongest and most common links between different genes and suicide. The expression studies enable a detailed description of gene expression patterns characteristic for different brain regions. In order to obtain reliable results, it is crucial to match the groups of studied subjects and controls according to age, sex, ethnic background and post-mortem intervals. A promising approach for gene expression studies is the use of oligonucleotide microarrays for the analysis of mRNA levels in the brain tissue. In order to ensure high dependability of the final results, several microarray RNA integrity indicators can be used, such as noise (RawQ) or consistent β-actin and glyceraldehyde-3-phosphate dehydrogenase (GAPDH) 5'/3' signal ratios. However, there is some controversy in relation to the idea of the housekeeping genes and, since the levels of the mRNA for β-actin and GAPDH have also been found to be dependent of cellular profiles and activation, there is a need for finding new methods of controlling the RNA integrity [29]. When analyzing the whole genome, the researchers inevitably face the problem of processing large amounts of complex information. It is therefore important to use reliable tools to summarize and functionally explore the collected data. Here, electronic data bases, as well as gene ontology and enrichment algorithms prove indispensable. Another important element is the implementation of sufficiently stringent significance criteria to identify differentially expressed genes biologically relevant to suicide. One of the commonly used tests is the combination of an ANOVA and subsequent t-tests, also referred to as Fisher protected least significant difference (LSD) test [28]. What is more, when running multiple tests it is always important to confirm the statistical significance using corresponding tools, for example the Bonferroni correction [30]. The analysis of gene ontology categories is one of the possibilities to organize the collected data and transparently present the role of the distinguished genes. Sequeira et al. [28] looked at patterns of gene expression in the limbic system of suicide victims to identify differentially expressed genes. The vast majority of expression changes were observed in the amygdala and hippocampus. Amongst the most strongly overrepresented genes where those involved in transcriptional regulation and metabolism: APLP2, BACE1, SYT4, ADCY8, GABRA1 and GABRB1. The identification of the adenylate cyclase 8 gene (ADCY8) confirms previous evidence of a possible involvement of the adenyl cyclase (AC) signaling system in the pathophysiology of suicide (see below). Interestingly, the findings are paralleled by the reports from animal studies: APLP2 knockout mice present with a reduction in both density and number of docked vesicles at the active zone [31], while SYT4 knockouts display reduced levels of anxiety and depression-like behaviour [32]. Sequeira and colleagues also approach a problem of a possible confounding effect on the gene expression by psychoactive drugs.

Genetic studies offer a unique possibility of comparing the effects of both long-term substance abuse (in subjects with documented chronic dependence) and acute substance exposure (in subjects without such history but with positive toxicological screening) on gene expression. The same research group performed gene expression analyses in 17 cortical and subcortical brain regions from suicide victims with and without major depression and controls [33]. They observed the highest number of suicide specific alterations in prefrontal cortical areas and hippocampus (HIP). With the use of the gene ontology analysis, alterations of synaptic neurotransmission and intracellular signaling were revealed. Among these, glutamatergic [34] and GABAergic related genes were globally altered. Thalmeier et al. [35] also performed a classification of the differentially expressed genes according to their biological function and statistical analyses of the data, using post-mortem orbitofrontal cortex of violent suicide victims and control subjects. As a result, they found nine particularly interesting transcripts (CDCA7L, CDH12, EFEMP1, MLC1, PCDHB5, PTPRR, S100A13, SCN2B, and ZFP36). The subsequent pathway analysis showed that the gene ontology categories 'central nervous system development', 'homophilic cell adhesion', 'regulation of cell proliferation' and 'transmission of nerve impulse' were significantly enriched. In a re-analysis of a large set of Affymetrix Human Genome U133A microarray data, Kim et al. [36] compared gene expression levels between suicide completers vs. non-suicide groups within two diagnostic groups, namely bipolar disorder and schizophrenia. Among bipolar samples, 13 genes and among schizophrenia samples, 70 genes were found to be differentially expressed. According to real-time quantitative PCR data, PLSCR4 and EMX2 were significantly down-regulated in the schizophrenia suicide completers, but not in patients with bipolar disorder. One of the genes found to be up-regulated in the bipolar group was gamma-amino butyric acid A receptor, α5 subunit (GABRA5), which, similarly to the finding of GABRA1 up-regulation by Sequeira et al. [28], indicates a possible involvement of the GABAergic neurotransmitter system in suicide. The authors concluded that, since the overlap of genes among the two diagnostic groups was small, a larger number of disorder-specific genes was found. This suggests that disorder-specific pathways dominate over common pathways at the molecular level. Sibille and colleagues [37] used DNA microarrays to conduct a large-scale gene expression analysis in two regions of the human prefrontal cortex obtained from post-mortem matched groups of subjects with major depression who died by suicide, and control subjects who died from other causes and were free from psychiatric disorders. Although the authors investigated molecular and cellular pathways potentially involved in depression and suicidal behaviour, and tested several hypotheses of disease pathology and of their putative molecular impact, including changes in single genes, the existence of subgroups of patients or disease subtypes, or the possibility of common biological pathways being affected in the disease process, no evidence for molecular differences that correlated with depression and suicide was found. As a result, the authors hypothesized that either the research methods they used were not sensible enough, or the pathology characteristic for depression was localized in other brain areas and possibly rather associated with post-transcriptional effects and/or changes in protein levels or functions, than with altered transcription in the prefrontal cortex (PFC) Table 1 provides an overview of the genes possibly involved in suicide.

**Table 1:** **Genes possibly involved in suicide**

Gene symbol	Description	Change	Location	Diagnosis	Reference
A2M	alpha-2-macroglobulin	upregulation	PFC	SCH	35
ABCG2	ATP-binding cassette, sub-family G(WHITE), member 2	upregulation	PFC	aSCH	35
ADCY8	adenylate cyclase 8 (brain)	upregulation	LS	MD	27
ADRA2A	adrenoceptor alpha 2A	higher frequency of the C1291G SNP	G	upregulated	73, 115
ADRA2B	adrenoceptor alpha 2B	higher frequency of the rs1018351 SNP	G	NC	116
AGT	angiotensinogen (serpin peptidase inhibitor, clade A, member 8)	downregulation	PFC	SCH	35
AGXT2L1	alanine-glyoxylate aminotransferase 2-like 1	downregulation	PFC	SCH	35
AHCYL1	S-adenosylhomocysteine hydrolase-like 1	downregulation	PFC	SCH	35
ALDH1L1	aldehyde dehydrogenase 1 family, member L1	downregulation	PFC	SCH	35
APLP2	amyloid beta (A4) precursor-like protein 2	upregulation	L	MD	27
APOE	Apolipoprotein E	downregulation	PFC	SCH	35
APOLD1	apolipoprotein L domain containing 1	downregulation	PFC	SCH	35
ATP1A2	ATPase, Na+/K+ transporting, alpha 2 (+) polypeptide	downregulation	PFC	SCH	35
ATP1B2A	ATPase, Na+/K+ transporting, beta 2 polypeptide	downregulation	PFC	SCH	35
BACE1	beta-site APP-cleaving enzyme 1	upregulation	LS	MD	27
BBOX1	butyrobetaine (gamma), 2-oxoglutarate dioxygenase 1	downregulation	PFC	SCH	35
BDNF	brain-derived neurotrophic factor	downregulatoin	PFC,	HIP NC	173, 174
C3	complement component 3	upregulation	PFC	SCH	35
CD74	CD74 molecule, major histocompatibility complex, class II invariant chain	upregulation	PFC	SCH	35
CDCA7L	cell division cycle associated 7-like	not characterised	PFC	NC	34
CDH12	cadherin 12, type 2 (N-cadherin 2)	not characterised	PFC	NC	34
CHI3L1	chitinase 3-like 1 (cartilage glycoprotein-39)	downregulation	PFC	BD	35
CLDN10	claudin 10	downregulation	PFC	SCH	35
COMT	catechol-O-methyltransferase	higher frequency of the val158met SNP	G	NC, *male suicides only*	125
CRH	corticotropin releasing hormone	upregulation	PFC	SCH	35
CSF1R	colony stimulating factor 1 receptor	upregulation	PFCS	CH	35
CX3CR1	chemokine (C-X3-C motif) receptor 1	upregulation	PFC	SCH	35
DARPP-32	protein phosphatase 1, regulatory (inhibitor) subunit 1B	downregulation	PFC	SCH	128
DDIT4	DNA-damage-inducible transcript 4	downregulation	PFC	SCH	35
DUSP6	dual specificity phosphatase 6	upregulation	PFC	SCH	35
EFEMP1	EGF-containing fibulin-like extracellular matrix protein 1	downregulation	PFC	SCH	35
EFEMP1	EGF containing fibulin-like extracellular matrix protein 1	not characterised	PFC	NC	34
EIF5A	eukaryotic translation initiation factor 5A	downregulation	PFC	SCH	35
EMX2	empty spiracles homolog 2 (Drosophila)	downregulation	PFC	SCH	34, 35
EMX2	empty spiracles homolog 2 (Drosophila)	downregulation	PFC	BD	35
EPHX1	epoxide hydrolase 1, microsomal (xenobiotic)	downregulation	PFC	SCH	35
EVC	Ellis van Creveld syndrome	higher frequency of the rs1383180 polymorhism	G	NC, *male suicides only*	26
FAM107A	family with sequence similarity 107, member A	downregulation	PFC	SCH	35
FGFR3	fibroblast growth factor receptor 3 (achondroplasia, thanatophoric dwarfism)	downregulation	PFC	SCH	35
FXYD1	FXYD domain containing ion transport regulator 1 (phospholemman)	downregulation	PFC	SCH	35
GABRA1	gamma-aminobutyric acid (GABA) A receptor, alpha 1	upregulation	LS	MD	27
GABRA5	gamma-aminobutyric acid (GABA) A receptor, alpha 5	upregulation	PFC	BD	34, 35
GABRB1	gamma-aminobutyric acid (GABA) A receptor, beta 1	upregulation	LS	MD	27
GLUL	glutamate-ammonia ligase (glutamine synthetase)	downregulation	PFC	SCH	35
HLA-DPA1	major histocompatibility complex, class II, DP alpha 1	upregulation	PFC	SCH	35
HLA-DRA	major histocompatibility complex, class II, DR alpha	upregulation	PFC	SCH	35
HSPB1	heat shock 27 kDa protein 1	downregulation	PFC	SCH	35
HTR2C	5-hydroxytryptamine (serotonin) receptor 2C, G protein-coupled	higher frequency of the Cys23Ser polymorhism	G	NC, *female suicides only*	84
ID4	inhibitor of DNA binding 4, dominant negative helix-loop-helix protein	downregulation	PFC	SCH	36
IL17RB	interleukin 17 receptor B	downregulation	PFC	SCH	35
ITGB4	integrin, beta 4	downregulation	PFC	SCH	35
ITPKB	inositol 1,4,5-trisphosphate 3-kinase B	downregulation	PFC	SCH	35
LAPTM5	lysosomal associated multispanning membrane protein 5	upregulation	PFC	SCH	35
LDLR	low density lipoprotein receptor	downregulation	PFC	BD	35
LOC645745	metallothionein 1H-like protein	downregulation	PFC	SCH	35
lTrkB	neurotrophic tyrosine kinase, receptor, type 2	downregulation	PFC,	HIP NC	171
METTL7A	methyltransferase like 7A	downregulation	PFC	SCH	35
MLC1	megalencephalic leukoencephalopathy with subcortical cysts 1	downregulation	PFC	SCH	35
MLC1	megalencephalic leukoencephalopathy with subcortical cysts 1	not characterised	PFC	NC	34
MT1E	metallothionein 1E (functional)	downregulation	PFC	SCH	35
MT1F	metallothionein 1F (functional)	downregulation	PFC	SCH	35
MT1G	metallothionein 1G	dcownregulation	PFC	SCH	35
MT1H	metallothionein 1H	downregulation	PFC	SCH	35
MT1M	metallothionein 1M	downregulation	PFC	SCH	35
MT1X	metallothionein 1X	downregulation	PFC	SCH	35
NAV2	neuron navigator	downregulation	PFC	BD	35
NDRG2	NDRG family member 2	downregulation	PFC	SCH	35
NOTCH2	Notch homolog 2 (Drosophila)	downregulation	PFC	SCH	35
NPY	neuropeptide Y	upregulation	PFC	SCH	35
NTRK2	neurotrophic tyrosine kinase, receptor, type 2	downregulation	PFC	SCH	35
NTSR2	neurotensin receptor 2	downregulation	PFC	SCH	35
P2RY13	purinergic receptor P2Y, Gprotein coupled, 13	upregulation	PFC	SCH	35
p75NTR (NGFR)	nerve growth factor receptor	higher frequency of the missense polymorphism (S205L)	G	MD	23
PCDHB5	protocadherin beta 5	NC	PFC	NC	34
PLSCR4	phospholipid scramblase 4	downregulation	PFC	SCH	34, 35
PLSCR4	phospholipid scramblase 4	downregulation	PFC	BD	35
PPAP2B	phosphatidic acid phosphatase type 2B	downregulation	PFC	SCH	35
PRKCI	protein kinase C, iota	upregulated	PFC	NC	150
PTPRR	protein tyrosine phosphatase, receptor type, R	downregulation	PFC	NC	34
RAB31	RAB31, member RAS oncogene family	downregulation	PFC	SCH	35
RHOBTB34	Rho-related BTB domain containing 3	downregulation	PFC	SCH	35
S100A13	S100 calcium binding protein A13	not characterised	PFC	NC	34
SAT1	spermidine/spermine N1-acetyltransferase 1	downregulation, higher frequency of the rs6526342 SNP	PFC	MD	186, 187, 188, 189, 190, 192
SCN2B	sodium channel, voltage-gated, type II, beta subunit	not characterised	PFC	NC	34
SDC2	syndecan 2 (heparan sulfate proteoglycan 1, cell surfaceassociated, fibroglycan)	downregulation	PFC	SCH	35
SDC4	syndecan 4 (amphiglycan, ryudocan)	downregulation	PFC	SCH	35
SLC14A1	solute carrier family 14 (urea transporter), member 1 (Kidd blood group)	downregulation	PFC	SCH	35
SLC1A3	solute carrier family 1 (glial high affinity glutamate transporter), member 3	downregulation	PFC	SCH	35
SLC25A23	solute carrier family 25 (mitochondrial carrier; phosphate carrier), member 23	upregulation	PFC	SCH	35
SLC4A4	solute carrier family 4, sodium bicarbonate cotransporter, member 4	downregulation	PFC	SCH	35
SLC6A3	solute carrier family 6 (neurotransmitter transporter, dopamine), member 3	higher frequency of the rs403636 SNP	G	NC	116
SLC7A11	solute carrier family 7, (cationic amino acid transporter, y+system) member 11	downregulation	PFC	SCH	35
SOX9 SRY	(sex determining region Y)-box 9	downregulation	PFC	SCH	35
SPON1	spondin 1, extracellular matrix protein	downregulation	PFC	SCH	35
STCH	stress 70 protein chaperone, microsome-associated, 60 kDa	upregulation	PFC	BD	35
SYT4	synaptotagmin IV upregulation LS MD 27 TIMP1 TIMP metallopeptidase inhibitor 1	downregulation	PFC	BD	35
TM4SF1	transmembrane 4 L six family member 1	downregulation	PFC	BD	35
TNFSF10	tumor necrosis factor (ligand) superfamily, member 10	upregulation	PFC	SCH	35
TP53BP2	tumor protein p53 binding protein, 2	downregulation	PFC	SCH	35
TPD52L1	tumor protein D52-like 1	downregulation	PFC	SCH	35
TPH2	tryptophan hydroxylase 2	higher frequeny of the rs1386494 SNP	G	NC	88
TRIM23	tripartite motif-containing 23	upregulation	PFC	BD	35
TUBB2B	tubulin, beta 2B	downregulation	PFC	SCH	35
TYROBP	TYRO protein tyrosine kinase binding protein	upregulation	PFC	SCH	35
VIL2	villin 2 (ezrin)	downregulation	PFCS	CH	35
WFS1	Wolfram syndrome 1 (wolframin)	higher frequency of the 611R/611R genotype	G	NC	21, 22
ZFP36	ZFP36 ring finger protein	not characterised	PFC	NC	34
ZHX2	zinc fingers and homeoboxes 2	downregulation	PFC	BD	35
ZIC1	Zic family member 1 (odd-paired homolog, Drosophila)	downregulation	PFC	BD	35

### Proteomics

The analysis of proteome, defined as an entity of all proteins of a cell, a tissue or an organism at a certain time and under exact defined conditions [38] , can help identify new suicide-associated proteins or protein modifications. The proteome is by definition highly dynamic and variable, i.e. easily influenced by a change in various intra- and extracellular processes. Similarly, the variety of factors implicated in the pathophysiology of suicide might predominantly affect the proteome rather than the more stable genome. It is important to remember that, since every single gene determines a multitude of gene products, in order to understand molecular processes in neuropsychiatric disorders, it is necessary to unravel signal transduction pathways and complex interaction networks which affect proteins, not only DNA and mRNA. Proteomics utilizes high-throughput mass spectrometric methods for protein identification that can help to reveal protein expression levels, posttranslational modifications and protein-protein interactions [39]. On the other hand, the use of proteomics to examine the modifications in protein expression also enables the identification of the new candidate genes for suicide. Thanks to an international initiative, the Human Proteome Organization (HUPO) was founded. HUPO is an international scientific organization representing and promoting proteomics through international cooperation and collaborations. Through ensuring public availability of collected data, HUPO supports the development of proteomics and fosters further research (http://www.HUPO.org). The field of proteomic studies of post-mortem brain tissue is still relatively unexplored. Schlicht et al. [40] used proteomic analysis by means of two-dimensional polyacrylamide gel electrophoresis (2D- PAGE) to compare protein patterns in the post-mortem PFC tissues of suicide victims and controls. They found three proteins, which were present exclusively in the suicide group. By means of mass spectrometry, they managed to identify these proteins as a phosphorylated isoform of glial fibrillary acidic protein (GFAP), manganese superoxidase dismutase (SOD2) and α crystallin chain B (CRYAB). GFAP is a component of glial filaments specific to astrocytes and was found up-regulated in reactive gliosis, as well as in several neurodegenerative disorders [41]. Interestingly, it is exactly the phosphorylated form of GFAP that has been implicated in the pathophysiology of psychiatric disorders [42]. SOD2 is a major antioxidant enzyme involved in the detoxification of superoxide radicals. Schlicht and colleagues propose to interpret their finding of SOD2 in the PFC of suicide subjects as a compensatory mechanism under the conditions of intensified oxidative stress. This corresponds well with the reports of elevated levels of this enzyme in different psychiatric disorders [34, 43–45]. On the other hand, a study by Pae et al. [46] failed to support an association of manganese superoxide dismutase (one of the antioxidant enzyme) gene polymorphism (MnSOD: Ala-9Val) with the development of mood disorders or their clinical parameters in the Korean population. Nevertheless, looking at the association between the oxidative stress and suicidality seems to be a new interesting direction of research.

The third protein Schlicht and colleagues found present only in suicidal victims, CRYAB, belongs to the low molecular heat shock proteins (small heat shock protein, sHsp). One of its important roles is the protection of intermediate filaments, such as GFAP in astrocytes, against aggregation and inactivation. The authors conclude that the expression of these three proteins in the PFC of suicide victims might indicate a possible existence of an intermitting component between glial function and suicidal behaviour. They suggest that the serotonergic system can indeed play such a role, especially because animal studies showed 5HT-sensitive astrocytes from 5-HT-depleted regions of rat brain revealed up-regulation of GFAP synthesis [47]. This hypothesis is consistent with many reports confirming an involvement of the serotonergic system in suicide (see below). Brunner et al. [48] performed proteomic analyses of the CSF in un-medicated patients with major depressive disorder with and without a history of suicide attempt. Two-dimensional gel electrophoresis revealed that suicide attempters differed from non-attempters in one protein with an approximate molecular weight of 33 kD and an isoelectric point of 5.2. The authors concluded that proteomic analysis of the CSF could be a promising non hypothesis-driven screening method for the detection of new candidate genes in neurobiological suicide research.

### Neurotransmitter systems

#### Serotonergic system

A vast amount of data documents a role of abnormalities in the serotonergic system in suicidal behaviour. Different elements of this complex system, including serotonin receptors, serotonin transporter and tryptophan hydroxylase, the rate-limiting enzyme in the synthesis of serotonin, have been researched in this respect. Quite understandably, numerous post-mortem studies have also focused on changes in the serotonergic system.

#### Serotonin receptors

Out of the 14 known different receptors for serotonin, the post-mortem studies have mainly focused on 5HT-1A, 5HT-1B, 5HT-1D, 5HT-2A, 5HT-2C, which have been proposed to play a role in depressive disorder, increased stress response, anxiety and suicidal behaviour. 5HT-1A serotonin receptors have been studied using autoradiographic methods and homogenate binding methods with 8-hydroxy-2-(di-n-propyloamino) tetralin (8-OH-DPAT) as the ligand. Stockmeier and colleagues [49] reported that the binding of [3H]8-OH-DPAT to serotonin-1A receptors was significantly increased in the midbrain dorsal raphe of suicide victims with major depression, as compared with control subjects. The density of 5HT-1A receptor binding sites in the PFC was also found increased in nonviolent suicide victims [50]. This result however conflicts with a number of other studies focused on the same receptors in the PFC, which showed no changes in this region [51–53]. However, a more recent study by Stockmeier et al. [54] has shown that, while there was no difference in the agonist-binding to 5HT-1A receptors between depressed patients and control subjects, the antagonist-binding was significantly decreased in outer layers of the PFC obtained from subjects diagnosed with major depressive disorder, the psychiatric diagnosis most common in suicide victims. Furthermore, Arango et al. [55] described differences in density of [3H]8-OH-DPAT binding between nonviolent suicide victims and control subjects in Brodmann areas 8 and 9. Interestingly, this different density was observed in male suicide victims only. Finally, an increase in 5HT-1A receptor binding sites in the CA1 area of the HIP of suicide victims compared to controls was described by Joyce and colleagues [56]. However, other studies reported no differences in hippocampal 5HT-1A receptor binding [52, 53]. Arraz et al. [51] observed a significant decrease in the 5HT-1A binding affinity in suicide victims who died as a result of an overdose of tricyclic antidepressants. They tried to explain this by the higher sensitivity of this binding site to the acute administration of TCA.

In summary, although the results of studies focusing on a role of 5HT1A receptors in suicidality are inconsistent, it appears that there may be an increase in their density in particular areas of the brain (raphe nuclei, Brodmann area 8 and 9). The serotonin 1B receptor has been suggested to play a role in suicide, aggression and substance abuse [57]. New et al. [58] reported an association between the G861C polymorphism in the coding region of the 5HT-1B receptor gene and susceptibility to suicide attempts in Caucasian subjects, while, concordant to the results of other studies based on mixed population [59, 60], no association was observed in the ethnically mixed overall sample. Another study [61], including two ethnically homogenous samples of German and Slavic origin, does not support the involvement of this 5HT-1B receptor polymorphism in suicide. In the above mentioned study Arraz et al. [51] looked also at the 5HT-1D serotonin receptor. A significant decrease in 5HT-1D binding affinity was found in depressed suicide victims, while the number of 5HT-1D binding sites showed a significant decrease in the non-depressed suicide victims. 5HT-2A, another serotonin receptor with a possible role in suicidality, can be labeled by several ligands, such as ketaserin, spiperon or lysergic acid diethyl amide (LSD), none of them completely specific. The use of nonspecific ligands can explain inconsistent results of studies focusing on this receptor. While the results of the first study by Stanley and Mann [62], showing increased 5HT-2A binding sites in frontal cortex (FC) of suicide victims, were later confirmed by further research [63–68], a number of other studies presented contradictory results with no difference [51, 53, 69–72] or, in one case, a decrease [73] in this receptor binding. In order to eliminate these discrepancies, Pandey et al. [66] examined both protein and mRNA expression of 5HT-2A receptors in several regions of the post-mortem brains of teenage suicide victims. They found that both protein and mRTA expression levels of these receptors were significantly increased in the PFC and the HIP, but not in the nucleus accumbens of suicide victims. These results were next confirmed by Escriba et al. [74]. Shelton et al. [75] reported 5HT-2A receptor protein expression in Brodmann area 10 obtained from major depressive disorder subjects, both suicide victims and not, to be increased in comparison to non-depressive subjects. Regarding the 5HT-2C receptor, a regulatory role in mood, appetite and sexual behaviour was suggested [76, 77]. Pandey et al. [78] determined the protein and mRNA expression of 5HT-2C receptors in the PFC Brodmann area 9, the HIP and choroid plexus of suicide victims and normal controls. They found higher protein expression in the PFC, but not in other brain regions of suicidal subjects. On the other hand, there was no significant difference in the mRNA expression in any of the studied regions. Since 5HT-2C receptor mRNA undergoes post-translational editing into two different receptor isoforms [79], a possible involvement of this process in the pathophysiology of suicidal behaviour was also suggested. Gurevich et al. [80] found that the pre-mRNA editing of the 5HT-2C receptors at the C' site was significantly increased, while the editing at the D site was decreased, and the C site showed a trend towards increased editing in the suicide victims with a history of major depression as compared with control subjects. Dracheva et al. [81] described the pre-mRNA editing of the 5HT-2C receptor in subjects with bipolar disorder or schizophrenia. Those of the subjects who died by suicide showed differences in editing localized in dorsolateral PFC, which were not present in subjects who died of natural causes. It has been proposed that 5HT2C-receptor-coupled signaling is altered in suicide victims due to modified G protein-coupling as a result of mRNA editing [82]. A suggested association between suicide and the single nucleotide polymorphism in the coding region of the 5HT-2C receptor gene, resulting in (Cys22Ser) substitution, although most probably causing functional differences between the two variants [83], has not been proved [84]. However, a significant association was observed between female suicide victims of Slovenian origin and the SN polymorphism 68G>C (Cys23Ser) in 5HT-2C receptor gene [85].

#### Tryptophan hydroxylase

Tryptophan hydroxylase is the rate limiting enzyme in the synthesis of serotonin. Post-mortem studies, indicated a greater density and number of TPH-immunoreactive (TPH-IR) neurons in the dorsal raphe nucleus [86] and higher TPH-IR in the dorsal, but not in the median raphe nucleus, in depressed suicide victims [87]. About ten years ago, two isoforms of TPH have been identified, TPH1 and TPH2, of which the latter is expressed primarily in the brain [88]. The isoforms are encoded by two genes located on different chromosomes. Haplotype and association studies of different SNPs suggest the involvement of the TPH2 gene in suicide. Zill et al. [89] reported a statistically significant association between the rs1386494 SNP of the TPH2 gene and suicide. They also found three TPH2 haplotypes which significantly differed in their distribution between suicide completers and control subjects. In the TPH1 gene in turn, there are two common polymorphisms on intron 7: A218C and A779C (originally classified as U and L for upper and lower band), which are in very high linkage disequilibrium. In one post-mortem study, the AA genotype was associated with higher TPH immunoreactivity and lower 5-HT2A binding in the PFC compared to other genotypes in both suicides and non-suicides, suggesting a regulatory role of this enzyme in the functioning of serotonergic system [90].

#### Serotonin transporter

The serotonin transporter, a target protein for a number of antidepressant drugs, including SSRIs, has also been a subject of post-mortem brain studies. Depressed suicides were shown to present with fewer serotonin transporters in the PFC (suicide or major depression), hypothalamus (suicide), occipital cortex (major depression) and brainstem (suicide and major depression) [90, 91]. Importantly, this PFC deficit in suicide victims appears localized to the ventromedial PFC (a brain region involved in willed action and decision-making), whereas major depression is associated with lower binding throughout the PFC [92]. On the other hand, Bligh-Glover et al. [93] showed a significant increase in [(3)H]paroxetine binding to serotonin transporters in the entire dorsal raphe nucleus, progressing from rostral-to-caudal levels in both normal control subjects and suicide victims with major depression. At comparable rostral-to-caudal levels, there were no significant differences in serotonin transporters found between depressed suicide victims and normal control subjects in either the entire dorsal raphe nucleus or its constituent subnuclei. One of the proposed causes of lower serotonin transporter binding in depression and suicide was the prevalence of a short variant (S allele) of functional promotor polymorphism of the transporter gene (5-HTTLPR). This theory however has not been confirmed by either post-mortem [92] or imaging studies [94, 95]. Zupanc et al. [96] reported no association between polymorphisms in serotonin transporter gene (5-HTT) (polymorphism LPR in promoter and VNTR in the second intron), as well as in different serotonin receptor genes (HTR): HTR1A (polymorphism -1019C>G), HTR1B (polymorphisms 861G>C and -161A>T), HTR1F (polymorphism -78C>T) and HTR2A (polymorphism -1420C>T), and completed alcohol-related suicide.

### Noradrenergic and dopaminergic system

#### Adrenergic receptors

Gross-Isseroff [73] and colleagues reported significantly lower binding to alpha 1-receptors in several brain regions of the suicide group as compared with matched controls. The decrease in receptor density was observed in portions of the PFC, in the temporal cortex (TC) and the caudate nucleus (CN). An increase in alpha 1-adrenergic binding in PFC was confirmed by Arango et al. [97]. Meana and Garcia-Sevilla [98], as well as González et al. [99] showed an increase in the number of alpha 2-adrenoreceptor agonist binding sites in the HIP and the FC of depressed suicide victims. Ordway et al. [100] and Ordway [101] described the same effect in locus coeruleus (LC), the principal source of brain norepinephrine. In contrast to those studies, Gross-Isseroff et al. [102] reported no significant, region-dependent alterations in the density of alpha 2-adrenergic receptors in brains of suicide victims as compared to matched controls. Since a few subtypes of adrenergic receptors were described and specifically localised in different parts of the brain (alpha1A, alpha1B and alpha1D as well as alpha2A, alpha2B and alpha2C) [103, 104], post-mortem studies provided a possibility to establish differences in receptor subtype locations, in their density and functioning, as well as in their role in sucidality. According to the study by De Paermentier et al. [105], who looked at adrenergic receptors in suicide victims in relation to treatment with antidepressants, the number of alpha1A- and alpha1D-adrenoceptors did not differ significantly between antidepressant-free or antidepressant-treated suicide subjects and controls. In antidepressant-free suicide subjects, the number of alpha2-adrenoceptors, however not alpha2A-adrenoceptors in particular, was significantly higher in the TC. In antidepressant-treated suicides, significantly lower numbers of alpha2-adrenoceptors were found in occipital cortex (OC) and HIP (and for alpha2A-adrenoceptors in the CN and the amygdala) compared to controls. On the other hand, Meana et al. [106] found both the density and affinity of alpha 2A-adrenoceptors in the high-affinity state to be increased, especially in the FC and the hypothalamus (HTH) of depressed suicides, a finding later confirmed by Callado and colleagues [107], who described a greater proportion of alpha2A-adrenoceptors in the high-affinity conformation in the FC of depressed suicide victims. Moreover, González-Maeso et al. [108] described an increase in alpha 2A- adrenoceptor sensitivity in the FC of depressed suicide victims. García-Sevilla et al. [109] reported an up-regulation of alpha 2A-adrenoceptors in the PFC (Brodmann area 9) of suicide subjects. One study reported an increase in biding to beta-adrenergic receptors in the PFC and the TC of the suicide victims [110]. Gurguis et al. [111] examined agonist affinity and coupling efficiency of beta-adrenergic receptors to Gs protein in the brains of ten suicide victims. They found no differences in the receptor densities in either the FC or HIP of suicide victims compared to controls, however, the preliminary results indicated beta-adrenergic receptors supercoupling in suicide victims in both brain regions. Following these findings, Little et al. [112] tested the hypothesis that the pineal beta-adrenergic binding is increased in depressed persons committing suicide, reflecting diminished noradrenergic input. They found no differences in pineal beta-adrenergic receptors between suicide subjects with major depression compared to the matched controls.

When comparing the density of beta-adrenoceptors with that of alpha2-adrenoceptors, Sastre et al. [113] found that, in brains of suicide victims, the ratio between alpha2- and beta-adrenoceptors (alpha2-full agonist sites/beta-sites) was greater compared to controls. A genetic study of alpha2A-adrenoceptors revealed – in concordance to the results of most autoradiographic and immunolabelling studies – an increased mRNA expression of the receptor gene in suicide subjects [74]. Sequeira et al. [114] looked at genetic variations of the alpha 2A-adrenergic receptor gene (ADRA2A) at four loci, including three in the promoter region (g-1800t, c-1291 g and the g-261a), and a potentially functional locus, N251K, which leads to an amino acid change (asparagine to lysine). They found no significant differences at the promoter loci in terms of allelic or genotypic distribution between suicide victims and controls. However, analysis of the functional polymorphism N251K revealed that the 251 K allele was only present among suicides, though only in three out of 110 cases. These results were not replicated either by Martín-Guerrero et al. [115] or by Fukutake et al. [116], although the latter study reported a significant association of the C-1291G SNP in the promoter region of the alpha 2A-adrenergic receptor gene with suicide in Japanese females. A link between the rs1018351 SNP in alpha 2B-adrenergic receptor gene and suicidal attempt was also reported [117]. Since different studies mentioned above confirm alterations in cortical beta- and alpha-adrenergic receptor binding, Arango et al. [118] studied specifically the pigmented neurons of the LC [119], which provide the noradrenergic innervations to the cerebral cortex. In the samples from the suicide group, they observed 23% fewer LC neurons and a 38% lower density of LC neurons when compared to control samples. This led them to a conclusion that altered brain noradrenergic neurotransmission in suicide victims may be due to fewer noradrenergic neurons in the LC.

#### Tyrosine hydroxylase, Catechol-O-methyltransferase

Tyrosine hydroxylase (TH) is the rate-limiting enzyme for catecholamine synthesis. Ordway and collegaues [100] found concentration levels of TH in LC to be elevated in the samples from suicide victims. Persson et al. [120] studied TH gene expression and observed a non-significant tendency towards a lower incidence of the TH-KI allele among suicide subjects compared to controls, although there was a significant association of the K3 allele in the subgroup of patients with adjustment disorder and suicide attempt. In other studies a trend for association [121] or no association [122] were reported. Biegon and Fieldust [123] found no difference between suicide victims and controls in the levels of the dopamine beta-hydroxylase (DBH, the last enzyme in the synthesis of neuroepinephrine) immunoreactivity or in the number of TH immunoreactive cells. Catechol-O-methyltransferase is an enzyme metabolizing noradrenaline in the synaptic cleft. COMT has been shown to affect cognition and personality traits as well as neuronal activation [124, 125], which suggest it can also play a role in the susceptibility to suicidal behaviour. The COMT gene has a common functional polymorphism, val158met. One post-mortem study found the Val allele less prevalent, and the heterozygote Val/Met more common in male suicide victims than in controls [126].

#### Dopaminergic system

Although there is not much data on the role of the dopaminergic system in suicidality, a reduction in the dopamine turnover (significantly lower concentration of dihydroxyphenylacetic acid) was observed in the CN, putamen (P) and nucleus accumbens (NA) in a post-mortem study of depressed suicide victims [127]. Allard and Norlén [128] reported no difference in Bmax and Kd of the [3H]WIN 35,428 ligand binding to dopamine uptake sites in the CN between the suicide group and controls. Since dopamine-and-cAMP-regulated neuronal phosphoprotein (32 kDa) (DARPP-32) is expressed in brain regions receiving dopaminergic projections, including the PFC, and is implicated in the pathophysiology of schizophrenia, Feldcamp et al. [129] determined the DARPP-32 gene expression in suicide victims with schizophrenia. They found a significant difference in gene expression levels between schizophrenia patients who died by suicide vs. other causes of death, as well as the between the schizophrenia group and controls.

#### Glutamatergic system

Based on a known adaptation of the NMDA receptor complex in the rodent cortex in response to chronic antidepressant treatment, Nowak et al. [130] hypothesized glutamatergic dysfunction could be involved in psychopathology underlying suicide. Consequently, they conducted an analysis of the glutamatergic receptors in post-mortem brain probes. They reported a reduced proportion of high affinity, glycine displaceable [3H]CGP-39653 binding to glutamate receptors in suicide victims in comparison to age- and post-mortem interval-matched controls. In contrast, neither the potency nor the maximum efficacy of glycine to inhibit [3H]CGP-39653 binding was altered in the FC of suicide victims compared to controls. The potency of glycine to inhibit [3H]5,7-dichlorokynurenic acid binding to the strychnine-insensitive glycine receptor or the specific binding of [3H]5,7-dichlorokynurenic acid did not differ between the two groups. Likewise, neither basal nor glycine- or glutamate enhanced non-equilibrium binding of [3H]dizocilpine was altered in suicide victims. Nevertheless, the findings confirm an involvement of the glutamatergic system in suicidality. To further support this, Nowak et al. [119] reported a statistically significant decrease in the potency of zinc to inhibit [(3)H]MK-801 binding to NMDA receptors in the hippocampal but not cortical tissue of suicide subjects. The authors proposed that the alteration in zinc interaction with NMDA receptors may be involved in the psychopathology underlying suicide attempts. However, other studies found no [131] or very little [132] evidence for a role of NMDA-binding sites in the pathophysiology of suicide. Freed and collegues [133] measured the non-NMDA excitatory amino acid receptors by means of analyzing the [3H]AMPA binding in the FC, the CN, and the NA of post-mortem human brain tissue samples. They found a pronounced increase in total AMPA binding in the CN in subjects that had committed suicide when compared to matched controls. An up-regulation of the binding of the AMPA receptor, was also reported in a group of suicide victims by Noga et al. [134]. Different factors interacting with the glutamatergic system should also be taken into account in suicide research. One interesting example is a study by Kalkman [135]. Based on the fact that the glycogen synthase kinase 3 (GSK3) inhibitor, lithium, which has a proven effect against suicide, increased the activity of glutamine synthetase in an animal experiment [136], he proposed that suicide might be prevented by centrally acting GSK3 inhibitors.

#### Other neurotransmitter systems

Cheetham et al. [137] studied GABA receptors by quantitating benzodiazepine (BZ) binding sites. They reported the number of BZ binding sites was significantly greater in the FC of the total group of depressed suicide subjects compared to controls, but did not differ in the TC. Vinod and collegues [138], who looked at the brain samples of subjects with a history of alcohol abuse, found the cannabinoid CB(1) receptor density in the dorsolateral PFC was higher in patient with alcohol addiction who committed suicide then in those who died of other causes. Western blot analysis confirmed a greater immunoreactivity of the CB(1) receptor in alcohol dependent suicide victims. The CB(1) receptor-mediated [(35)S]GTP gamma S binding also indicated a greater signaling in this group. The same group reported an upregulation of CB1 receptors and agonist-stimulated [35S]GTPgammaS binding in the PFC of depressed suicide victims [139].

Table 2 summarizes the findings regarding neurotransmitter systems and suicidality.

**Table 2:** **Neurotransmitter systems and suicide**

System element			Change	Location
SEROTONERGIC	Receptors	5HT-1A	upregulated	RN, FC
		5HT-1D	downregulated	PFC
		5HT-2A	downregulated	PFC, HIP, FPC
		5HT-2C	upregulated	PFC
		5-HT4	upregulated	FC, HIP, CN, AN
	Enzymes	TPH	upregulated	RN
	Transporter	Serotonin transporter	downregulated	PFC, RN
NORADRENERGIC	Receptors	Alpha-1	downregulated	PFC, TC, CN
		Alpha-2	upregulated in drug-free subjects	HIP, FC, LC, HTH
			downregulated in antidepressant- treated subjects	OC, HIP
		Alpha-2A	upregulated in drug-free subjects	EC, HTH, PEE
			downregulated in antidepressanttreated subjects	AN, CN
		Beta	upregulated	PFC, TC
		Alpha-2 ‘Beta ratio	increased	G
	Enzymes	TH	upregulated	LC
DOPAMINERGIC	Dopamine turnover	Dihydroxy- phenylacetic acid concentration	decreased	CN, P. NA
GLUTAMATERGIC	Receptors	NMDA	downregulated	G
			Zinc inhibition decreased	HIP
		AMPA	upregulated	CN
GABA-ERGIC	Receptors	GABA	upregulated	FC
ENDOCANNABINOID	Receptors	CB(1)	upregulated	PFC

### Cell-signalling research

#### Protein kinases A and C

A disturbance in a signal-transduction process from cell surface receptors to the nucleus was postulated as a one of the mechanisms underlying suicidal behaviour. As a result, a possible role of protein kinase A, an enzyme involved in the adenyl cyclase (AC) signalling pathway, as well as protein kinase C, a component of the phosphoinositide (PI) signalling system, were investigated. Protein kinase A (PKA), activated by cyclic AMP, works by phosphorylisation of several intracellular proteins and activates certain transcription factors. Two different PKA izoenzymes were identified, known as PKAI and PKAII, both compromised of R and C units. The I and II PKA isoenzymes are built of different R subunits (called RI and RII), each of them consisting of RIα and RIβ or RIIα and RIIβ subunits respectively. Furthermore, three C subunits, known as Cα, Cβ and Cγ, were described. Post-mortem studies showed 3[H]-cAMP binding and PKA activity to be significantly decreased in the PFC of suicide victims [140, 141]. Moreover, a decrease in the catalytic activity of PKA in Brodmann area 9 and the HIP was also observed in suicide subjects [142]. In concordance with this observation, Dwivedi and colleagues [141] reported the protein and mRNA expression of PKA subunits RIIβ and Cβ to be decreased in PFC of suicide subjects when compared with controls. Pandey et al. [143], who looked at the activity of the PKA subunits in the post-mortem brain of teenage suicide victims, found a decreased cAMP binding and PKA activity in the PFC, but not in the HIP. Shelton and colleagues [75] studied 5-HT (2A) receptor abundance and PKA activity in post-mortem brain tissue specimens from persons with a history of major depression. They reported an increased 5-HT(2A) receptor abundance and decreased PKA activity in the depressed sample. 5-HT(2A) receptor availability was significantly inversely correlated with PKC activity in controls, but not with PKA activity in the depressed sample. The authors propose these abnormalities of 5-HT(2A) receptor abundance may depend on receptor uncoupling and heterologous regulation by PKA. A study of serotonin 5-HT2A and 5-HT4 binding parameters and their second messengers 1,4,5-inositol triphosphate (IP3) and cyclic adenosyl monophosphate [129] in the FC, HIP, CA and amygdala of suicide victims by Rosel et al. [72] revealed significantly higher number of 5-HT4 receptors and higher second messenger cAMP concentrations in the FC and CN of the depressed suicide victims as compared with the control group. Furthermore, significantly increased 5-HT2A binding sites and IP3 concentrations were noted in the CN of the suicide group, together with a significantly reduced number of 5- HT2A binding sites, higher binding affinity and increased IP3 concentrations in the HIP. Dwivedi et al. [144] studied Rap-1, one of the major substrates of protein kinase A, involved in neuroprotection and synaptic plasticity, which can be directly activated by cAMP through exchange proteins (Epac1, Epac2). They found that Rap-1 activation was significantly reduced in the PFC and the HIP of the suicide group. This was associated with significant reductions in Rap-1 messenger RNA and protein levels. In contrast, protein level of only Epac-2, but not Epac-1 was significantly increased in the PFC and the HIP of these subjects. To further support an involvement of the AC signalling system in suicidal behaviour, a response to the beta(1)-adrenoceptors agonist-stimulated AC activity was found significantly lower in post-mortem brain samples from subjects with major depressive disorder [145].

Protein kinase C is a regulatory enzyme involved in the modulation of many neuronal and cellular functions, such as neurotransmitter synthesis and release, regulation of receptors and ion channels, neuronal excitability, gene expression, secretion and cell proliferation [146]. The PKC family has been sub-grouped into three classes, namely conventional, novel and atypical izoenzymes, which differ in molecular structure and enzymatic activity [147–149]. Pandley et al. [150] found that the binding of 3[H]phorbol 12,13-dibutyrate by PKC was significantly decreased in both membrane and cytosol fractions obtained from the PFC of teenage suicide victims. Both in the PFC and in the HIP of teenage suicides, the PKC activity was decreased, so was the protein expression of conventional isoenzymes. The decreased activity was reflected by significantly decreased mRNA levels for those isoenzymes. Choi et al. [151] found expression levels of the PRKCI gene, encoding atypical iota isoenzyme of the PKC family, increased in the PFC of suicide victims as compared to non-suicide controls. One of the substrates for phosphorylation by protein kinase C, myristoylated alanine-rich C kinase substrate (MARCKS), involved in neurotransmitter release and re-uptake, was postulated to play a role in mood disorders [152]. According to another study by Pandey and colleagues [153], protein levels of MARCKS were significantly increased in the membrane fraction of the PFC and the HIP obtained from depressed suicide subjects as compared to normal controls. The PKC-mediated MARCKS phosphorylisation was also determined, and found significantly decreased in the membrane fraction of the PFC and the HIP obtained from both depressed and non-depressed suicide subjects. The authors suggest a decrease in MARCKS phosphorylisation may be a common feature of suicide victims, independent of the psychiatric diagnoses.

#### Transcription factor CREB

Activation of PKA, as well as PKC, phosphorylates several transcription factors, which then influence gene expression. Cyclic AMP response element binding protein (CREB) is a transcription factor, which has been implicated in the pathophysiology of suicide. Odagaki et al. [154] quantified the level of CREB in the PFC of depressed suicide victims by Western blotting. They observed a significant increase in the levels of CREB, both in total and phosphorylisated forms in brains of depressed victims compared to those of control subjects. The increase was observed specifically in antidepressant-free subjects, but not in the antidepressant-treated subjects, which suggests the role of cAMP signaling system in the therapy with antidepressants. Young and colleagues [155] reported increased numbers of phosphorylated CREB stained cells in several amygdalar nuclei in subjects who had died by suicide. On the other hand, Dwivedi et al. [142] reported the protein expression of CREB was significantly decreased in the nuclear fractions of both the PFC and the HIP obtained from suicide victims compared with control subjects. This was reflected by a decrease in the mRNA levels of CREB, as well as in the CRE-DNA binding activity, as measured in the nuclear fractions of both the PFC and the HIP. In accordance with these findings, Pandey and colleagues [156] reported a specific decrease in CRE-DNA binding and the mRNA as well as protein expression of CREB in the PFC of teenage suicide victims. However, they did not find any significant differences in the HIP CREB levels between teenage suicides and controls.

#### Cytokines

Cytokines are small cell-signaling protein molecules which serve as immuno-modulating agents. Changes in the immune system have been suggested to play a role in depression and suicidality, following observations of a negative influence of IFN-alpha on patients' mood [157, 158], as well as of altered levels of pro- and anti-inflammatory cytokines in the serum [159–161] or the CSF of depressed patients [162, 163]. What is more, Lindqvist et al. [164] found IL-6 level in CSF was significantly higher in suicide attempters than in healthy control subjects. The number of post-mortem studies focusing on cytokines is limited. However, Steiner et al. [165] found microgiliosis, one of the elements of immunological activation of the brain tissue, to be increased in the post-mortem brains of suicide victims diagnosed with affective disorders and schizophrenia. Tonelli and colleagues [166] described increased mRNA expression of IL-4 and IL-3 in the orbitofrontal cortex of female victims of suicide, as well as of IL-13 in PFC of male suicide victims compared with control subjects. Pandey et al. [167] reported significantly increased mRNA and protein expression levels of IL-1β, IL-6, and TNF-α in Brodmann area 10 of teenage suicide victims as compared with normal control subjects Table 3 summarizes the findings regarding the role of cell-signalling in suicide.

**Table 3:** **Cell-signalling in suicide**

System element			Change	Location
Adenylate cyclase	Protein kinase A	Enzyme activity	decreased	PFC, HIP
		Enzyme subunits RIl, C3 levels	decreased	PFC, *adult suicides*
		Enzyme subunits Rkt, R113 levels	decreased	PFC, *teenage suicides*
		Enzyme substrate Rap- 1	reduced activation	PFC, HIP
	Second messenger	cAMP level	increased	PC, CN
	cAMP effector	Epac-2 level	increased	PFC, HIP
		CREB level	decreased	PFC, HIP
			increased	PFC, *antidepressants-free subjects*
			increased density of CREB stained cells	AN
Phospholipase C	Protein kinase C	Enzyme activity	decreased	PFC, HIP, *teenage suicides*
		Enzyme substrate MARCKS	increased concentration, decreased phosphorylation	PFC, HIP
	Second messenger	IP3 level	increased	HIP, CN
Cytokines	Interleukins	IL-1β level	increased	FC, teenage suicide
		IL-3 level	increased	PC, *female suicides*
		IL-4 level	increased	FC, *female suicides*
		IL-6 level	increased	PC, teenage suicide
		IL-13 level	increased	PC, *male suicides*
	TNF-Family	TNF-α level	increased	PC, teenage suicide

### Neural plasticity

#### Neurotrophic factors

Neurotrophic factors are a family of proteins that induce the survival, development, and functional differentiation of neurons [168], which includes among others nerve growth factor (NGF), brain-derived neurotrophic factor (BDNF), glial cell line-derived neurotrophic factor (GDNF), its family ligands neurturin (NTN), persephin (PSP), and artemin (ARTN), neurotrophin-3 (NT-3), and neurotrophin-4 (NT-4). The expression of BDNF is regulated by CREB through Ca2+ and the cAMP response element within exon 3 of BDNF. Given that a change in CREB levels has been shown in the post-mortem brains of suicide victims (see above), and the treatment with antidepressants causes an increase of BDNF in the brain [169], it has been hypothesized that depression and/or suicide may be associated with a decreased level of BDNF. Furthermore, neurotrophic factors have been discussed in the etiology of several other neuropsychiatric disorders such as schizophrenia and dementia [170, 171]. Dwivedi and colleagues [172] determined the protein and mRNA expression levels of BDNF in the PFC and the HIP, finding them both to be significantly decreased in suicide victims compared with controls. According to Karege et al. [173], the levels of BDNF and NT-3 in the HIP and PFC of medication-free suicidal subjects were significantly decreased in comparison with controls. Interestingly, in suicide victims treated either with antipsychotic or antidepressant drugs, neurotrophine levels were not significantly different from those in non-suicide controls. To further support a role of BDNF in suicidality, two genetic studies, including a study by Sarchiapone et al. [174], as well as a meta-analisys by Zai an colleagues [175], implicate an association between functional SNP (rs6265) of the BDNF gene and suicidal behaviour. The SNP results in (Val) to (Met) substitution in the proBDNF protein at codon 66, and is thought to be associated with low BDNF levels in psychiatric disorders. In contrast, de Luca and colleagues [176], who analyzed the BDNF Val66Met polymorphism in suicide completers and the parent-of origin effect (POE) in suicide attempters, found no evidence for allelic imbalance or POE of BDNF for suicidal behaviour. Dwivedi et al. [172] measured the protein and mRNA expression of the TrkB receptor, a member of a family of tyrosine kinases with the highest affinity to the binding of BDNF. Out of the two known isoforms, the protein and mRNA expression levels of the full-length the TrkB receptors, but not their truncated isoform, were found significantly decreased in the PFC and in the HIP of suicide subjects. Ernst and colleagues [177] looked at a truncated splice variant of the TrkB (TrkB.T1), the only isoform expressed in astrocytes under normal conditions. They found that 10 out of 28 examined brain probes from suicide completers demonstrated significant decreases of TrkB.T1 in the Brodmann areas 8 and 9, a result not accounted for by substance abuse comorbidity or by reduction in astrocyte number. Moreover, it was found that the methylation of 2 CpG dinucleotides, sites 2 and 5 of the TrkB promoter, is associated with the decreased expression of TrkB.T1.

The levels of the whole blood glial cell line-derived neurotrophic factor (GDNF) has been shown to be decreased in remitted patients with mood disorders [178] and one post-mortem study showed an increase in GDNF concentration in the PC of patients with depressive disorder [44], therefore suggesting its involvement in the pathophysiology of mood disorders and suicidality. Furthermore, both human and animal model have shown that different antidepressants cause an acute activation of protein tyrosine kinase (PTK) and extracellular signal-regulated kinase (ERK), leading to the activation of CREB and resulting in an increase of the GDNF production [179–181]. Otsuki et al. [182] used qRT-PCR in peripheral blood cells of patients with major depressive and bipolar disorders to measure the expression levels of mRNAs of different neurotrophic factors. They reported a reduced expression of GDNF, ARTN, and NT-3 mRNAs in patients with current major depressive disorder. Altered expressions of these mRNAs were found neither in remitted patients with depressive disorder, nor in bipolar patients.

#### Polyamins

Polyamins, including putrescine, spermidine and spermine, are low molecular weight aliphatic amines involved in cell proliferation, apoptosis, immunity and oxidative stress response [183–185]. Changes in the expression of the polyamines and their metabolic enzymes have been postulated in different mental disorders, including suicidal behaviour [186]. Alterations in the expression of polyamine-related genes were identified using post-mortem brain tissue from suicide completers. Sequiera et al. [187] looked at the spermine/spermidine N(1)- acetyltransferase gene (SSAT), encoding the rate-limiting enzyme in the catabolism of polyamines. They found that an allele of a single nucleotide polymorphism (SNP) rs6526342 showed higher frequency among suicide cases. The authors suggest this SNP allele, located in the SSAT polyamine- responsive element regulatory region (SSAT342A/C), which demonstrates a significant effect on SSAT brain expression levels, may increase the predisposition to suicide. To further support this, Fiori et al. [188] showed that suicide completers who possessed the haplotype containing the risk allele for the very same SNP (rs6526342) demonstrated decreased SAT1 expression in Brodmann areas 4, 8/9 and 11. They also identified three other polymorphisms - an insertion/deletion (rs6151267), and two SNPs (rs6526342 and rs928931) - located in the promoter region of SAT1, which were found to be involved in the regulation of gene expression and, according to the authors, might thus provide a mechanism for the decreased SAT1 expression observed in suicide completers. A reduction in spermine/spermidine N(1)-acetyltransferase gene expression in cortical regions of individuals who have died by suicide was confirmed by Klempan et al. [189] and Guipponi et al. [190]. The latter study does not support a role of the rs6526342 SNP in suicide, but highlights the importance of epigenetic factors influencing the level of SAT1 expression, a conclusion drawn also by Fiori and Turecki [191]. Global gene expression profiling of the polyamine system in suicide completers by Fiori and colleagues [192] identified 14 genes displaying differential expression. These included spermidine/spermine N1-acetyltransferase, spermine oxidase, spermine synthase, S-adenosylmethionine decarboxylase, ornithine decarboxylase antizymes 1 and 2, arginase II, aldehyde dehydrogenase 3 family, member A2, brain creatine kinase, mitochondrial creatine kinase 1, glycine amidinotransferase, glutamic- oxaloacetic transaminase 1, and arginyl-tRNA synthetase-like genes. Since many of these genes displayed altered expression across several brain regions, the authors concluded that a dysregulated polyamine metabolism is a widespread phenomenon in the brains of suicide completers. In another study, Fiori et al. [193] genotyped 1255 French-Canadian individuals for the 63 polymorphisms, spread across 1074 four polyaminergic genes. The group was followed-up longitudinally for 22 years. The authors found an association between suicide attempts and polymorphisms in SAT1 and the OATL1 genes. Additionally, different genetic variants of SAT1 were also associated with anxiety and mood disorders, conditions linked to suicidal behaviour. Taking into consideration a considerable amount of evidence linking changes in the polyamine metabolism with suicide, Chen et al. [194] hypothesised levels of spermine, spermidine and putrescine would be different in the brains of suicide victims. To this end, they used a GC-MS method developed specifically for the measurement of spermidine and putrescine levels in post-mortem brain tissue [195], to compare the probes derived from suicide completers and controls. In correlation with the conclusions drawn from the previous genetic studies, both putrescine and spermidine levels were found significantly elevated in the brain of suicide victims with major depression.

Table 4 gives an overview of the changes in neural plasticity observed in suicide victims.

**Table 4:** **Changes in neural plasticity in suicide**

System element			Change	Location
Neurotrophic factors	Neurotrophins	BDNF level	decreased	PFC, HIP,*antidepressants-free subjects*
		NT-3 level	decreased	PFC, HIP, *antidepressants-free subjects*
	BDNF-binding receptor	TrkB receptor concentration	decreased	PFC, HIP
		TrkB.T1 truncated form receptor concentration	decreased	PFC
Polyamins	Putrescine levels		increased	G
	Spermidine levels		increased	G

#### Neuroendocrinology

Abnormalities in HPA axis have been postulated both in mood disorders and suicide. On the one hand, most patients with depression have been shown to present with hypercortisolemia both in plasma and CSF, increased cortisol response to adrenocorticotropic hormone (ACTH) and a deficient feedback mechanism, reflected in an abnormal dexamethasone suppression test [11] as well as by enlarged pituitary and adrenal glands [196–198]. On the other hand, a large amount of evidence linking HPA axis dysfunction to suicidality has been published to date. Yerevanian et al. [199] found that DST non-supressors with unipolar depression were significantly more likely to commit suicide than DST suppressors. Similar results were reported by Coryell and Schlesser [200] in a 15-years long follow-up study of patients with major depressive disorder or a depressed type of schizoaffective disorder. As for suicide attempts, some studies report an association with DST non-suppression [201, 202], while others do not [203, 204]. This suggests HPA axis disturbances may be more strongly related to suicide completion rather than just attempts. Thus, studying the elements of this axis in brain probes from suicide subjects is another interesting area. Raadsheer et al. [205] reported that the mean total number of CRH-expressing neurons and of CRH neurons co-expressing AVP in hypothalamic paraventricular nucleus (PVN) was significantly higher in depressed subjects than in the control group. Merali et al. [206, 207] found CRH-immunoreactivity levels among suicides were elevated in the LC and in frontopolar, dorsolateral prefrontal (DMPFC) and ventromedial prefrontal cortices, but reduced at the dorsovagal complex (DVC). Austin et al. [208] hypothesized that CRH levels in suicide victims would be increased also beyond the HTH, in specific brainstem regions. They next tested this with help of radioimmuno-cytochemistry, using primary antiserum to CRH and a ( [125])I-IgG secondary antibody. They reported an increased level of CRH-immunoreactivity in the LC, in the median raphe and in the caudal dorsal raphe of depressed suicide subjects compared to controls. In conditions of chronic hypersecretion of CRH, due to down-regulation, a reduced number of CRF receptor binding sites should be present. Accordingly, Nemeroff and colleagues [209] described a marked reduction in the number of CRF binding sites in the FC of suicide victims compared with controls. Subsequently, the mRNA levels of CRH1, but not CRH2 receptors for corticotropin were found decreased in suicide brains [206]. What is more, a shift in CRH1:CRH2 mRNA ratio was also reported in pituitaries of suicide subjects [210]. The feedback regulation of the HPA axis by glucocorticosteroids is mediated through two intracellular receptors, known as mineralocorticoid (MRs) and glucocorticoid (GRs) receptors. GRs, which are believed to play an important role in the regulation of stress response, have been studied in the peripheral tissues of depressed patients [211], who present with a reduced GRs function sometimes referred to as GR resistance, as well as in post-mortem brains of patients with different psychiatric disorders [212, 213], which show decreased levels of GRs in comparison to normal controls. The above described findings indicate that there is a need to also examine a possible role of GRs in the pathology of suicide. To date, one study by Lopez and colleagues [214] focused on the localization of glucocorticoid receptor (GR) mRNA and pro-opiomelanocortin (POMC) mRNA in anterior pituitaries of suicide subjects. The analysis of the corticotrophic cell clumps showed that the suicide victims had a higher POMC mRNA density per cell and a larger corticotrophic cell size than controls, however no differences in GR mRNA were detected between the two groups. McGowan et al. [215] reported that the expression of total glucocorticoid receptor mRNA was significantly reduced in suicide victims with a history of childhood abuse when compared to suicide victims who did not suffer childhood abuse or to controls. Supriyanto et al. [216] tested a possible association between suicide and polymorphisms of the glucocorticoid receptor (NR3C1), the mineralocorticoid receptor (NR3C2), and the FK506 binding protein 5 (FKBP5) genes, all of them involved in HPA axis regulation. The authors found that the distributions of TT, TC, and GT haplotypes of the FKBP5 gene (comprised of rs3800373 and rs1360780) in completed suicide and control groups were significantly different. Since the FK506 binding protein 5 interacts functionally with mature corticoid receptor hetero-complexes, this confirms a crucial role of the HPA axis in suicidality. However, no significant differences in genotypic distribution of any single SNP in the three genes in question were observed between the individuals who completed suicide and control groups Table 5.

**Table 5:** **HPA axis in suicide**

System element			Change	Location
Hormones	CRH levels		increased	LC, DLPFC, FPC, VMPFC, CN
			decreased	DVC
	POMC mRNA level		increased	aPIT
Receptors	CRH-receptors	receptor density	decreased	FC
	CRH1-receptor mRNA level	decreased	G	CRH1-receptor mRNA level
	CRH1/CRH2receptor ratio	decreased	PIT	CRH1/CRH2receptor ratio

## Discussion

### Limitations

Post-mortem studies on brain tissue have a number of limitations. One of them is the relatively short period of time, within which the samples should be collected. For example, the levels of dihydropyrimidinase-related protein-2 were found to decrease within 6 h after death [217]. Thus, the post-mortem interval should ideally not exceed 6 hours, which sometimes can be very hard to achieve. Another problem is the small sample size, which significantly limits the possibility to generate data fit for detailed statistical analysis, especially regarding genetics. Only further improvement in brain collection programs can help to confirm the results of previous pilot studies based on small groups of subjects. The issue is further complicated by an considerable overlap between psychiatric disorders, all of which might alone be associated with certain neurobiological abnormalities, and suicide. With the limited availability of post-mortem samples from psychiatric patients who died of natural causes, it is very difficult to examine if the neurobiological abnormalities in the post-mortem brains are related to suicidality or rather to the psychiatric diagnoses. Hence, there is a need for comparison studies of samples obtained from suicide victims with a particular diagnosis, and those taken from subjects with exactly the same diagnosis, who died of natural causes. Another important problem is the influence of pharmacological and other therapies used in psychiatric patients on brain biology. Thus, it is difficult to distinguish neurobiological factors independently associated with suicidality and not related to comorbid psychiatric disorders or caused by psychiatric treatment. Last but not least, the role of obtaining a very detailed psychological history should never be underestimated. The use of validated methods to reconstruct psychiatric history by means of extensive proxy-based interviews [218] is indispensable in order to eliminate possible confounding variables influencing the results, such as the history of childhood abuse, other trauma, personality disorders, but also somatic diagnoses.

### Future possibilities

Post-mortem studies of suicidality provide many possibilities for widening our knowledge of the underlying neuropathology and of the influence of medication and substance abuse, as well as for constructing and testing different clinical hypotheses. Several interesting new research directions of post-mortem studies are conceivable for the future, including innovative genetic and epigenetic studies with the use of new data bases and new brain collection programs. Another unique possibility is the comparison of neurobiological factors characteristic for suicide in the samples representing particular psychiatric disorders, as described above. The same goes for differentiating between neurobiological abnormalities related to suicidal behaviour and those associated with completed suicide. Another interesting possibility is the description of the effects of different psychiatric drugs, their actions on genes expression, protein levels and neuro-receptors, and their efficacy in preventing suicide. Last but not least, the impact of chronic or acute substance intoxication on the brain neurobiology and its influence on the suicide risk could also be further researched in post-mortem studies.

## Conclusions

Studies on post-mortem brain tissue provide numerous possibilities for a deeper insight into the neurobiology of different psychiatric disorders, including suicidal behaviour. With new methods being now introduced to improve the reliability of results, genetic information being openly available for analyses and comparisons and the brain tissue banks being founded and expanded, the post-mortem studies are likely to grow in number and further develop in future.

## Abbreviations

AN: Amygdalar nuclei
BD: Bipolar disorder
CN: Caudate nuceus
DLPFC: Dorsolateral prefrontal cortex
DVC: Dorsovagal complex
FC: Frontal cortex
FPC: Frontopolar cortex
G: Global
HIP: Hippocampus
HTH: Hypothalamus
LC: Locus coeruleus
LS: Limbic system
MD: Major depression
NA: Nucleus accumbens
NC: Not characterised
OC: Occipital cortex
P: Putamen
PIT: Pituitary
aPIT: Anterior pituitary
PFC: Prefrontal cortex
RN: Raphe nuclei
SCH: Schizophrenia
TC: Temporal cortex
VMPFC: Ventromedial prefrontal cortex.
